# Delineating an Epigenetic Continuum for Initiation, Transformation and Progression to Breast Cancer

**DOI:** 10.3390/cancers3021580

**Published:** 2011-03-29

**Authors:** Kang Mei Chen, Josena K. Stephen, Usha Raju, Maria J. Worsham

**Affiliations:** 1 Department of Otolaryngology/Head and Neck Surgery, Henry Ford Hospital, 1 Ford Place, 1D, Detroit, MI 48202, USA; E-Mails: kchen1@hfhs.org (K.M.C.); jstephe2@hfhs.org (J.K.S.); 2 Department of Pathology, Henry Ford Hospital, Detroit, 1 Ford Place, 1D, Detroit, MI 48202, USA; E-Mail: uraju1@hfhs.org

**Keywords:** benign, premalignant, transformation, DNA methylation, progression, continuum

## Abstract

Aberrant methylation of promoter CpG islands is a hallmark of human cancers and is an early event in carcinogenesis. We examined whether promoter hypermethylation contributes to the pathogenesis of benign breast lesions along a progression continuum to invasive breast cancer. The exploratory study cohort comprised 17 breast cancer patients with multiple benign and/or *in situ* lesions concurrently present with invasive carcinoma within a tumor biopsy. DNA from tumor tissue, normal breast epithelium when present, benign lesions (fibroadenoma, hyperplasia, papilloma, sclerosing adenosis, apocrine metaplasia, atypical lobular hyperplasia or atypical ductal hyperplasia), and *in situ* lesions of lobular carcinoma and ductal carcinoma were interrogated for promoter methylation status in 22 tumor suppressor genes using the multiplex ligation-dependent probe amplification assay (MS-MLPA). Methylation specific PCR was performed to confirm hypermethylation detected by MS-MLPA. Promoter methylation was detected in 11/22 tumor suppressor genes in 16/17 cases. Hypermethylation of *RASSF1* was most frequent, present in 14/17 cases, followed by *APC* in 12/17, and *GSTP1* in 9/17 cases with establishment of an epigenetic monocloncal progression continuum to invasive breast cancer. Hypermethylated promoter regions in normal breast epithelium, benign, and premalignant lesions within the same tumor biopsy implicate *RASSF1*, *APC*, *GSTP1*, *TIMP3*, *CDKN2B*, *CDKN2A*, *ESR1*, *CDH13*, *RARB*, *CASP8*, and *TP73* as early events. DNA hypermethylation underlies the pathogenesis of step-wise transformation along a monoclonal continuum from normal to preneoplasia to invasive breast cancer.

## 1. Introduction

Progression from a pre-malignant lesion to malignancy is associated with cytogenetic and molecular genetic aberrations [1-3]. Benign breast lesions (BBD) with clinically significant pre-malignant potential include atypical ductal hyperplasia (ADH), atypical lobular hyperplasia (ALH), and lobular carcinoma *in situ* (LCIS) [4] whereas ductal carcinoma in situ (DCIS) is considered a pre-invasive malignant lesion [5].

Epigenetics is the regulation of changes in gene expression by mechanisms that do not involve changes in DNA sequence. Establishment and maintenance of epigenetic control (gene silencing) has several aspects, which include promoter region hypermethylation, methyl-binding proteins, DNA methyltransferases, histone deacetylases and chromatin state. Epigenetic alterations, in particular promoter hypermethylation, are proving to be consistent and early events in neoplastic progression [6-9]. Such alterations are thought to contribute to the neoplastic process by transcriptional silencing of tumor suppressor gene expression and by increasing the rate of genetic mutation [10,11].

The potential that aberrant methylation may be pharmacologically reversible offers additional treatment opportunities for breast cancer. We examined whether promoter hypermethylation contributes to the pathogenesis of benign and premalignant breast lesions along a progression continuum to invasive breast cancer.

## 2. Results and Discussion

For the 17 breast cancer cases, the number of multiple lesions within a breast cancer biopsy ranged from 2–7. DNA was obtained from 71 lesions, and included 15 normal, 16 benign, 11 carcinoma*-in-situ* (CIS) and 29 tumor lesions (from 68 tumor blocks), which were interrogated for methylation status using the Methylation-Specific Multiplex Ligation Dependent Probe Amplification (MS-MLPA) assay (Table 1).

The MS-MLPA assay was performed in all cases and MS-MLPA results were confirmed by Methylation Specific PCR (MSP) for those cases 1, 2, 3, and 5 with sufficient DNA for bisulfate conversion (Table 1).

### 2.1. The Methylation-Specific Multiplex Ligation Dependent Probe Amplification (MS-MLPA) Assay results

**Figure 1.**
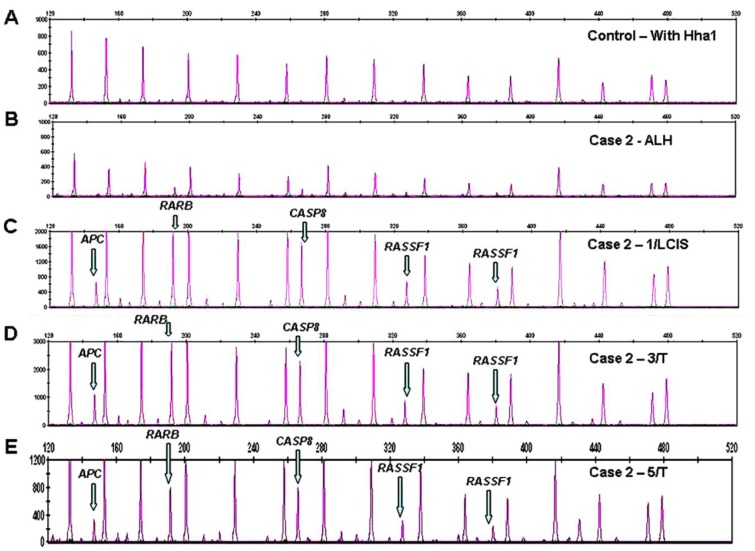
Methylation-Specific Multiplex Ligation Dependent Probe Amplification (MS-MLPA) probe mix with *Hha*I enzyme in normal (control) DNA (**A**), and 4 biopsy specimens from Case 2 (**B-E**). Note methylation of *APC, RARB, CASP8* and *RASSF1* (both *RASSF1* probes) in biopsies LCIS, tumor block #3 and tumor block # 5(**C-E**). (ALH – atypical lobular hyperplasia, LCIS – lobular carcinoma *in situ*, 3T – block 3 tumor and 5T – block 5 tumor).

Promoter methylation was detected in 11/22 tumor suppressor genes in 16/17 cases. DNA hypermethylation of *RASSF1* was most frequently present in 14/17 cases with establishment of an epigenetic progression continuum to invasive breast cancer in 7 of the 14 cases, and an early transformation event along a progression continuum from normal to benign to breast cancer in Cases 3 and 5, benign (ALH) to LCIS to tumor in Case 2 (Figure 1 C-E), and normal to DCIS to tumor in Case 11 (Table 1), respectively.

Aberrant methylation of *APC* was noted in 12/17 cases with evidence of a progression continuum from normal to benign to invasive breast cancer in Case 3, benign to CIS to tumor in Case 2 (Figure 1C-1E), benign (papilloma) to tumor in Case 5, and DCIS to tumor in Cases 9, 15, and 18. Promoter hypermethylation of *GSTP1* was observed in 9/17 cases with a progression continuum from normal to benign to invasive breast cancer in Case 5 and benign to invasive breast cancer in Cases 3 and 7. Methylation of *TIMP3* in 4/17 cases linked normal and benign (ALH) lesions in Case 7, and DCIS and tumor lesions in Case 15. Aberrant methylation of *CDKN2B* and *ESR1* in 4/17 cases, connected benign (ALH) and tumor lesions in Case 7. *RARB*, *CDH13*, *CASP8*, *CDKN2A*, were less frequently methylated, connecting CIS and tumor lesions in Case 2 (*RARB*, *CDH13*, *CASP8*), Case 18 (*CDH13*), and Case 15 (*CDKN2A*), Table 1.

### 2.2. Methylation Specific PCR (MS) Results

**Figure 2.**
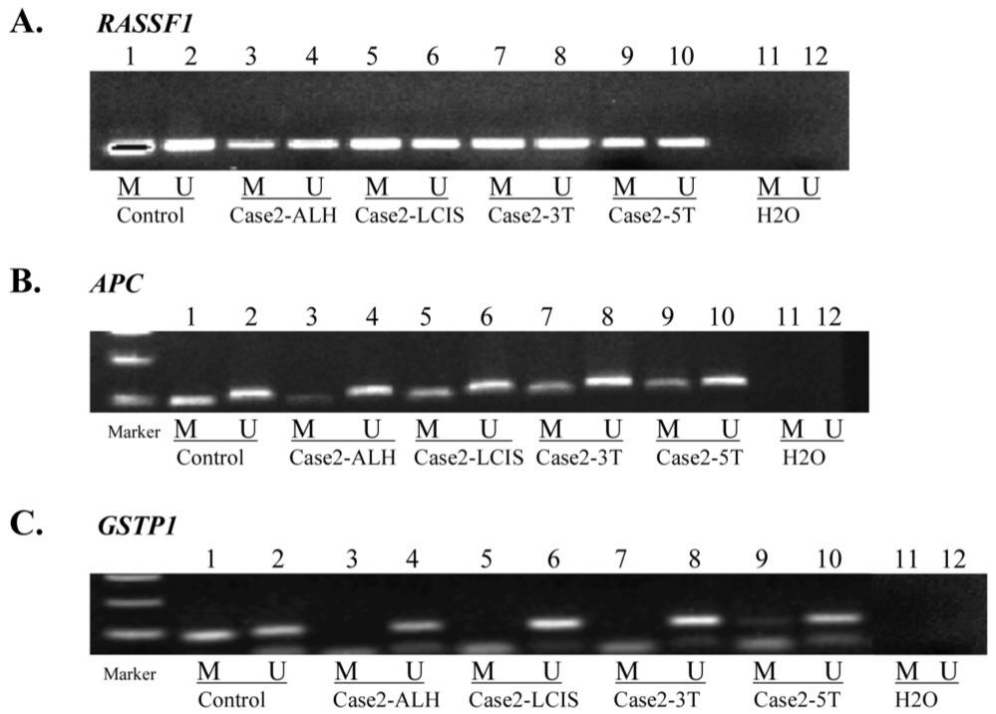
Methylation Specific PCR (MSP) confirmation of aberrant methylation detected by MS-MLPA for *RASSF1, APC,* and *GSTP1* for Case 2. (**A**) (*RASSF1*): Lanes 1-2: universal methylated and unmethylated controls. Lanes 3-10 span biopsies ALH – 5T. Note presence of methylated product in all biopsies. Lanes 11-12: negative control. (**B**) (*APC*): Lanes 1-2: universal methylated and unmethylated controls. Lanes 3-10 span biopsies ALH – 5T. Note presence of methylated product in all biopsies. Lanes 11-12: negative control. (**C**) (*GSTP1*): Lanes 1-2: universal methylated and unmethylated controls. Lanes 3-10 span biopsies ALH – 5T. Note presence of methylated product in biopsy 5T. Note absence of methylated product in biopsies ALH, LCIS and 3T. Lanes 11-12: negative control. (ALH – atypical lobular hyperplasia, LCIS – lobular carcinoma *in situ*, 3T – block 3 tumor and 5T – block 5 tumor).

MSP for *APC, GSTP1* and *RASSF1*, performed in Cases 1, 2, 3 and 5 (Table 1, Figure 2) confirmed aberrant methylation of *APC* detected by MS-MLPA for Case 1 (tumor), Case 2 (LCIS and tumor) and Case 3 (tumor block 6 and 10).

In addition, MSP indicated aberrant methylation of *APC* in Case 1 (DCIS), Case 2 (ALH), Case 3 (normal, hyperplasia, and papilloma) and Case 5 (papilloma and tumor blocks 2 and 8), not detected by MS-MLPA (Table 1). MSP confirmed aberrant methylation of *GSTP1* for Case 3 (tumor block 10) and lack of methylation in Case 5 tumor block 2, indicated by MS-MLPA. Additionally MSP identified *GSTP1*methylation in Case 2 (tumor block 5) and Case 3 (hyperplasia and tumor block 6), not detected by MS-MLPA. In Case 1 (DCIS) and Case 5 (normal, fibroadenoma, papilloma, and tumor block 8), MSP did not confirm MS-MLPA results for *GSTP1*. MSP of *RASSF1* confirmed methylation detected by MS-MLPA for Case 1 (tumor), Case 2 (LCIS and tumor blocks 3 and 5), Case 3 (tumor blocks 6 and 10) and Case 5 (fibroadenoma and papilloma). Additional *RASSF1* methylation was detected by MSP in Case 2 (ALH), Case 3 (normal and hyperplasia) and Case 5 (normal and tumor block 2).

### 2.3. Discussion

Epigenetic alterations produce heritable changes in gene expression without a change in the DNA coding sequence itself. Promoter region hypermethylation is known to be an early event in carcinogenesis [12-15]. The consequence of CpG island hypermethylation, especially for those islands associated with tumor suppressor gene promoters is the loss of tumor suppressor function, which contributes to tumorigenesis [2]. Clonal epigenetic alterations in benign and precancerous lesions may reflect biological peculiarities pertinent to tumor behavior.

Recurrent genomic aberrations are good indicators of genes that are causally associated with cancer development, transformation or progression. Our previous studies [14,15] have demonstrated that epigenetic events of DNA hypermethylation underlie the pathogenesis of benign sinonasal and laryngeal papillomas, including establishing a monoclonal origin for recurrent respiratory papillomas (RRP).

This study underscores promoter hypermethylation as an early event in the pathogenesis of breast cancer. Aberrant methylation in normal breast epithelium or benign lesions from four cases with progression implicated *TP73*, *TIMP3*, *CDKN2B*, *ESR1*, *APC*, *GSTP1* and *RASSF1* as early events. *RASSF1* was most frequently methylated (14/17) cases followed by *APC* (12/17) cases and *GSTP1* in 9/17 cases.

Methylation of *RASSF1A* and *APC* was reported to occur more frequently in benign samples from high risk women (determined by the Gail model) than in samples from low or intermediate risk women and was associated with epidemiologic markers of increased breast cancer risk [16]. *RASSF1A* methylation has been highly correlated with breast cancer risk, atypical cytology and benign breast disease requiring biopsy [17]. With respect to age, *RASSF1A* methylation has been noted to increase linearly between ages 32 and 55 [17]. *RASSF1A* influences the G1-S cell cycle checkpoint by regulating the level of cyclin D1 protein [18]. Methylation of *RASSF1A* leads to accumulation of cyclin D1 and may represent one mechanism for overriding cell cycle control under conditions of increased cell cycle pressure [16]. In microdissected breast tissue, Lehmann *et al.* [19] showed that the *RASSF1A* promoter was methylated in all epithelial hyperplasia and papilloma samples and in 83% of ductal carcinoma samples *in situ*, suggesting methylation of *RASSF1A* as a new marker for nonphysiological epithelial proliferation in the breast [19]. The study also found that in most cases of progression to invasive growth, epigenetic inactivation takes place before invasive growth develops, an observation confirmed by Pasquali *et al.* [20] who observed a progressive gain of methylation for *RASSF1A* from normal to hyperplasia acquiring statistical significance at CIS and invasive carcinoma.

Genetic and epigenetic alterations in *APC* (adenomatosis polyposis coli), a tumor suppressor gene originally implicated in colon cancer have been reported in other malignancies including breast cancers. A study of 76 breast cancer patients by Liu *et al.* [21] demonstrated that *APC* gene methylation correlated positively with TNM staging and negatively with protein expression suggesting a role in the development of breast cancer. Lee *et al.* [22] reported methylation of the *APC* promoter 1A in 42% of breast cancer aspiration fluid samples. They also found that *APC* was unmethylated in the aspiration fluids from normal breast tissue in patients with breast cancer and all benign breast disease patients in their cohort.

Glutathione S-transferase pi (*GSTP1*), at chromosome 11q13 [23], encodes for the glutathione S-transferase pi enzyme and plays an important role in detoxification and in susceptibility to cancer and other diseases. The pi-class of glutathione S-transferase enzymes has been associated with preneoplastic and neoplastic changes [24]. Inactivation of *GSTP1* by promoter hypermethylation is characteristic of steroid related neoplasms such as breast, liver, and prostate cancers [24,25]. *GSTP1* had increased levels of methylation in carcinoma *in situ* and invasive carcinoma samples [20] and MSP-based studies of human tissues demonstrated that *GSTP1* promoter methylation is associated with gene inactivation in about 30% of primary breast carcinomas [24]. In our cohort, nine of 17 cases demonstrated aberrant methylation of *GSTP1* and in three cases (Cases 3, 5, 7) a transformation continuum from benign to invasive carcinoma was evident.

In this study hypermethylation of *TIMP3* was observed in normal and benign (ALH) lesions in Case 7 and in DCIS and tumor lesions in Case 15, implicating *TIMP3* as an early event. *TIMP3* belongs to a family of molecules that inhibit the proteolytic activity of matrix metalloproteinases [26,27]. *TIMP3* is methylated in ∼30% of human breast cancer cell lines as well as ∼30% of primary breast tumors [28].

For cases with sufficient DNA, MSP for the most part confirmed promoter hypermethylation detected by MS-MLPA. MSP did not confirm MS-MLPA methylation of *GSTP1* observed in several biopsies for Cases 1 and 5. While a distinct advantage of MS-MLPA is the ability to examine aberrant promoter methylation in multiple cancer genes in a single assay run, multiplex PCR of a large number of gene probes (22 unique genes) inherently encounters competitive amplification. In contrast, MSP examines only one gene at a time [29] and therefore, is more sensitive than MS-MLPA [29]. Additionally, MS-MLPA methylation and quantitation detection algorithms may miss hypermethylation events that do not reach the threshold for detection [2]. Regardless, MS-MLPA profiling of multiple genes for aberrantly methylated promoter regions is a valuable screening tool to determine frequency and pattern of gene inactivation in tumorigenesis. These epigenetic signatures, upon subsequent validation as diagnostic or prognostic epigenetic biomarkers, can become reduced to a more definitive candidate gene panel of only a few key genes. The latter would be amenable for increased detection sensitivity by a targeted 3 or 4 MS-MLPA gene probe panel or by MSP alone.

Tumor markers are often biochemical surrogates for tumor presence [30]. Serum carcinoembryonic antigen (CEA) concentrations are used clinically to monitor the tumor burden in patients with advanced breast cancer [31]. Given the lack of circulating tumor markers, particularly tumor associated antigens, and a dearth of reliable genetic markers for diagnosis of early breast cancer [30], methylation changes, which often precede apparent malignant changes, have potential utility in the early diagnosis of cancer [32].

## 3. Experimental Section

### 3.1. Patient Cohort

The study cohort comprised 17 breast cancer patients (Table 1) with concurrently present tumor, normal breast epithelium when present, and one or more benign lesions histologically characterized as fibroadenoma, hyperplasia, sclerosing adenosis, apocrine metaplasia, papilloma, atypical lobular hyperplasia (ALH), atypical ductal hyperplasia (ADH), or *in situ* lesions of lobular (LCIS) or ductal carcinoma (DCIS). Normal, benign, CIS, and tumor tissue were obtained from separate blocks when available and when present within the same tissue block, were microdissected to prevent contamination and separate out individual lesions for DNA extraction. Normal breast tissue controls for methylation assays were obtained from women without breast cancer who underwent reduction breast surgeries. The study was undertaken according to approved institutional review board protocols.

### 3.2. DNA Extraction

Whole 5 micron formalin-fixed tissue sections (5 whole sections) or micro-dissected (10 sections) tumor, normal breast epithelium, benign, and in situ lesions were processed for DNA extraction as previously described [33].

### 3.3. The Methylation-Specific Multiplex Ligation Dependent Probe Amplification (MS-MLPA) Assay

The Multiplex Ligation-Dependent Probe Amplification assay allows for the relative quantification of approximately 41 different DNA sequences in a single reaction requiring only 20 ng of human DNA. The standard use of the technique to observe quantitative changes in copy number [3,34-36] and the adaptation of the MLPA to detect aberrant methylation (MS-MLPA) has been detailed elsewhere [2,14,29,37].

The probe design is similar to ordinary MLPA probes. For 26/41 probes, the recognition sequence detected by the MLPA probe is contained within a restriction site for the methyl-sensitive enzyme, *Hha*I. The 41 gene probe panel (Table 2) interrogates 35 unique genes implicated in cancer including breast cancer for losses and gains in a separate reaction in the absence of the methyl-sensitive enzyme *Hha*I.

**Figure 3.**
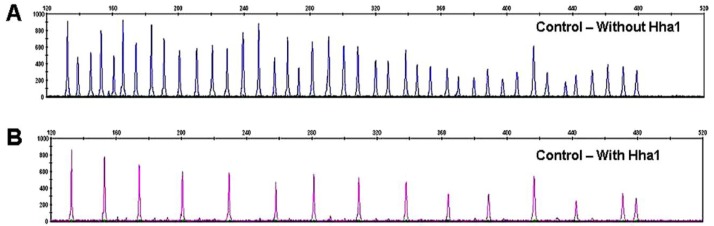
Normal (control) MS-MLPA assay results of MS-MLPA probe mix without (**A**) and with (**B**) *Hha*I enzyme. Note 15 methylation control peaks in the control DNA sample (**B**).

Because there are two probes each for *MLH1, RASSF1* and *BRCA2*, a normal control DNA sample will generate 41 individual peaks in the absence of *Hha*I (Figure 3A). A concurrently run reaction with the 41 gene probe set in the presence of *Hha*I is designed to detect aberrant promoter hypermethylation by taking advantage of a *Hha*I site in the promoter region of 22 of the 35 unique genes (note that one of the two *BRCA2* probes is designed to recognize a region outside the *Hha*I recognition site, Table 2). Fifteen of the 41 gene probes are designed outside a Hha1 site and serve as undigested controls (Figure 3B). Upon digestion of the sample DNA with *Hha*I, probes that recognize the unmethylated regions will not generate a signal because these sequences have become cut by *Hha*I and cannot bind to the probe. Conversely, a MLPA probe will bind to an intact methylated site, spared by *Hha*I, and generate an amplification signal (Figure 1, C-E).

Aberrant methylation is identified as the appearance of a signal peak that is otherwise absent in normal DNA samples (Figure 1, C-E). To quantify whether one, both, or more copies of a specific gene locus becomes aberrantly hypermethylated, a previously described mathematical algorithm was employed [2].

### 3.4. Bisulfate Modification and Methylation-Specific Polymerase (MSP) Chain Reaction Assay

For cases with sufficient DNA for bisulfate modification, methylation by MS-MLPA was confirmed by MSP for a limited number of genes (Table 3). Genomic DNA (100ng) from formalin-fixed paraffin embedded breast lesion tissue and control universal methylated DNA (Chamicon International, Inc) and control unmethylated DNA (normal genomic DNA) were modified using the EZ DNA methylation gold kit (Zymo Research, Orange, CA, USA) during which methylated DNA is protected and unmethylated cytosine is converted to uracil [29]. The modified DNA served as a template using primers specific for the methylated or modified unmethylated sequences (Table 3).

MSP amplification was performed using 3 μL of bisulfite modified DNA in a final volume of 25 μL PCR mix containing 1× PCR buffer, 2.5 mM dNTP, 1 mM MgCl2 and 1 U Amp gold Taq DNA polymerase, 0.5 μM primer followed by 38 cycles at 95 °C 45 seconds, 62 °C 45 seconds, 72 °C 1 min [29]. The resultant PCR products were separated on 2% agarose gel stained with ethidium bromide and visualized under UV illumination.

## 4. Conclusions

In this study, aberrant methylation of *RASSF1, APC* and *GSTP1* were both frequent as well as early events in the progression continuum from normal to benign to invasive cancer and support a monoclonal transformation continuum to breast cancer progression.

Identifying epigenetic alterations in a precancerous lesion may lead to the discovery of biomarkers that add to the knowledge of risk assessment and early detection, and may provide molecular targets for chemopreventive interventions. Because promoter hypermethylation is potentially reversible, molecules that regulate the methylation status of DNA are considered promising targets for new cancer therapies.

## Figures and Tables

**Table 1.** Cases with a methylation continuum.

**Case 1**	4/N	3/DCIS	2/IDC		
		APC*	APC*		
		*GSTP1*	RASSF1*		

**Case 2**	5/N	4/ALH	1/LCIS	3/ILC	5/ILC
		*APC*†	APC*	APC*	APC*
		*RASSF1*†	RASSF1*	RASSF1*	RASSF1*
			*RARB*	*RARB*	*RARB*
			*CASP8*	*CASP8*	*CASP8*
			*CDH13*	*CDH13*	
					*GSTP1*†

**Case 3**	11/N	3/H	5/Pap	6/IDC	10/IDC
	*APC†*	*APC*†	*APC*†	APC*	APC*
	*RASSF1*†	*RASSF1*†		RASSF1*	RASSF1*
		*GSTP1*†		*GSTP1*†	GSTP1*
	*CDKN2B*				*CDH13*
	*ESR1*				*TP73*

**Case 5**	6/N	5/FA	5/Pap	2/IDC	8/IDC
			*APC*†	*APC*†	*APC*†
	*RASSF1*†	RASSF1*	RASSF1*	*RASSF1*†	
	*GSTP1*	*GSTP1*	*GSTP1*		*GSTP1*
	*TP73*				*TP73*
		*CDKN2A*			*ESR1*

**Case 7**	7/N	2/ALH	4/ILC	5/ILC	
		*GSTP1*	*GSTP1*		
	*TIMP3*	*TIMP3*			
		*CDKN2B*	*CDKN2B*		
		*ESR1*	*ESR1*		
			*RASSF1*		

**Case 9**	1/N	4/DCIS	2/IDC	3/IDC	
		*APC*	*APC*	*APC*	
		*RASSF1*	*RASSF1*	*RASSF1*	
			*RARB*	*RARB*	
				*CDKN2B*	

**Case 11**	10/N	1/DCIS	4/DCIS	3/IDC	8/IDC
	*RASSF1*	*RASSF1*	*RASSF1*	*RASSF1*	*RASSF1*
	*TIMP3*				

**Case 15**		4/DCIS	10/IDC		
		*APC*	*APC*		
		*TIMP3*	*TIMP3*		
		*CDKN2A*	*CDKN2A*		
		*GSTP1*	*RARB*		

**Case 18**	3/N	4/DCIS	3/IDC	4/IDC	
		*APC*	*APC*	*APC*	
		*RASSF1*	*RASSF1*	*RASSF1*	
		*CDH13*	*CDH13*	*CDH13*	
			*GSTP1*	*GSTP1*	

**Case 19**		8/DCIS	5/IDC		
		*RASSF1*	*RASSF1*		
			*APC*		
			*TIMP3*		

**M** = methylated by MS-MLPA; M* = MS-MLPA methylation confirmed by MSP; **M^†^** = methylation detected by MSP only; N = normal; FA = fibroadenoma; H = hyperplasia; Pap = papilloma; AM = apocrine metaplasia; AM = apocrine metaplasia; ADH = atypical ductal hyperplasia; ALH = atypical lobular hyperplasia; SA = sclerosing adenosis; LCIS = lobular carcinoma in situ; DCIS = ductal carcinoma *in situ*; IDC = invasive ductal carcinoma; ILC = invasive lobular carcinoma; number refers to tissue block

**Table 2.** Methylation-Specific MLPA Probe Panel (ME001).

#	Gene probe	Chrom Loc	#	Gene probe	Chrom Loc	#	Gene probe	Chrom Loc
1	***TP73***	01p36		*CDK6*	07q21.3		*PAH*	12q23
2	***CASP8***	02q22.3	12	***CDKN2A***	09p21	21	*CHFR*	12q24.33
3	***VHL***	03p25.3	13	***CDKN2B***	09p21	22	***BRCA2***	13q12.3
4	***RARB***	03p24	14	***DAPK1***	09q34.1		*BRCA2*	13q12.3
5	*MLH1	03p21.1		*AI651963*	10p14		*MLH3*	14q24.3
6	***MLH1***	03p21.1		*CREM*	10p12.1		*TSC2*	16p13.3
	*CTNNB1*	03p22	15	***PTEN***	10q23.3		*CDH1*	16q22.1
7	*RASSF1	03p21.3	16	***CD44***	11p12	23	***CDH13***	16q24.2
8	***RASSF1***	03p21.3	17	***GSTP1***	11q13	24	***HIC1***	17p13.3
9	***FHIT***	03p14.2	18	***ATM***	11q23	25	***BRCA1***	17q21
	*CASR*	03q21	19	***IGSF4***	11q23		*BCL2*	18q21.3
10	*APC*	05q21		*TNFRSF1A*	12p13		*KLK3*	19q13
11	***ESR1***	06q25.1		*TNFRSF7*	12p13	26	*TIMP3*	22q12.3
	*PARK2*	06q26	20	***CDKN1B***	12q13.1			

Bold = probes with *Hha*I site (n = 26 probes);

* genes with multiple probes in the promoter region.

**Table 3.** Methylation and Unmethylation MSP Primer Sequences for Breast lesions.

**Gene**	**Methylation Specific Primers**	**Unmethylation Specific Primers**	**Size**
***APC-F***	5′-TATTGCGGAGTGCGGGTC	5′-GTGTTTTATTGTGGAGTGTGGGTT	M-97 bp
***APC-R***	5′-TCGAAGAACTCCCGACGA	5′-CCAATCAACAAACTCCCAACAA	U-108 bp
***GSTP1-F***	5′-TTCGGGGTGTAGCGGTCGTC	5′-GATGTTTGGGGTGTAGTGGTTGTT	M-91 bp
***GSTP1-R***	5′-GCCCCAATACTAAATCACGACG	5′-CCACCCCAATACTAAATCACAACA	U-97 bp
***RASSF1-F***	5′-GGGTTTTGCGAGAGCGCG	5′-GGTTTTGTGAGAGTGTGTTTAG	M-169 bp
***RASSF1-R***	5′-GCTAACAAACGCGAACCG	5′-CACTAACAAACACAAACCAAAC	U-169 bp

F = forward, R = reverse

## References

[1] Worsham M.J., Pals G., Raju U., Wolman S.R. (2002). Establishing a molecular continuum in breast cancer DNA microarrays and benign breast disease. Cytometry.

[2] Worsham M.J., Chen K.M., Meduri V., Nygren A.O., Errami A., Schouten J.P., Benninger M.S. (2006). Epigenetic events of disease progression in head and neck squamous cell carcinoma. Arch. Otolaryngol. Head Neck Surg..

[3] Worsham M.J., Pals G., Schouten J.P., Van Spaendonk R.M., Concus A., Carey T.E., Benninger M.S. (2003). Delineating genetic pathways of disease progression in head and neck squamous cell carcinoma. Arch. Otolaryngol. Head Neck Surg..

[4] Arpino G., Laucirica R., Elledge R.M. (2005). Premalignant and in situ breast disease: Biology and clinical implications. Ann. Intern. Med..

[5] Worsham M.J., Raju U., Lu M., Kapke A., Cheng J., Wolman S.R. (2007). Multiplicity of benign breast lesions is a risk factor for progression to breast cancer. Clin. Cancer Res..

[6] Hanahan D., Weinberg R.A. (2000). The hallmarks of cancer. Cell.

[7] Warnecke P.M., Bestor T.H. (2000). Cytosine methylation and human cancer. Curr. Opin. Oncol..

[8] Yang X., Yan L., Davidson N.E. (2001). DNA methylation in breast cancer. Endocr. Relat. Cancer.

[9] Widschwendter M., Jones P.A. (2002). DNA methylation and breast carcinogenesis. Oncogene.

[10] Wajed S.A., Laird P.W., DeMeester T.R. (2001). DNA methylation: An alternative pathway to cancer. Ann. Surg..

[11] Jones P.A., Baylin S.B. (2002). The fundamental role of epigenetic events in cancer. Nat. Rev. Genet..

[12] Gasco M., Sullivan A., Repellin C., Brooks L., Farrell P.J., Tidy J.A., Dunne B., Gusterson B., Evans D.J., Crook T. (2002). Coincident inactivation of 14-3-3sigma and p16ink4a is an early event in vulval squamous neoplasia. Oncogene.

[13] Nuovo G.J., Plaia T.W., Belinsky S.A., Baylin S.B., Herman J.G. (1999). In situ detection of the hypermethylation-induced inactivation of the p16 gene as an early event in oncogenesis. Proc. Natl. Acad. Sci. USA.

[14] Stephen J.K., Vaught L.E., Chen K.M., Shah V., Schweitzer V.G., Gardner G., Benninger M.S., Worsham M.J. (2007). An epigenetically derived monoclonal origin for recurrent respiratory papillomatosis. Arch. Otolaryngol. Head Neck Surg..

[15] Stephen J.K., Vaught L.E., Chen K.M., Sethi S., Shah V., Benninger M.S., Gardner G.M., Schweitzer V.G., Khan M., Worsham M.J. (2007). Epigenetic events underlie the pathogenesis of sinonasal papillomas. Mod. Pathol..

[16] Lewis C.M., Cler L.R., Bu D.W., Zochbauer-Muller S., Milchgrub S., Naftalis E.Z., Leitch A.M., Minna J.D., Euhus D.M. (2005). Promoter hypermethylation in benign breast epithelium in relation to predicted breast cancer risk. Clin. Cancer Res..

[17] Euhus D.M., Bu D., Milchgrub S., Xie X.J., Bian A., Leitch A.M., Lewis C.M. (2008). DNA methylation in benign breast epithelium in relation to age and breast cancer risk. Cancer Epidemiol. Biomarkers Prev..

[18] Shivakumar L., Minna J., Sakamaki T., Pestell R., White M.A. (2002). The rassf1a tumor suppressor blocks cell cycle progression and inhibits cyclin d1 accumulation. Mol. Cell Biol..

[19] Lehmann U., Langer F., Feist H., Glockner S., Hasemeier B., Kreipe H. (2002). Quantitative assessment of promoter hypermethylation during breast cancer development. Am. J. Pathol..

[20] Pasquali L., Bedeir A., Ringquist S., Styche A., Bhargava R., Trucco G. (2007). Quantification of cpg island methylation in progressive breast lesions from normal to invasive carcinoma. Cancer Lett..

[21] Liu Z., Yang L., Cui D.X., Liu B.L., Zhang X.B., Ma W.F., Zhang Q. (2007). Methylation status and protein expression of adenomatous polyposis coli (APC) gene in breast cancer. Ai Zheng.

[22] Lee A., Kim Y., Han K., Kang C.S., Jeon H.M., Shim S.I. (2004). Detection of tumor markers including carcinoembryonic antigen, apc, and cyclin d2 in fine-needle aspiration fluid of breast. Arch. Pathol. Lab. Med..

[23] Moscow J.A., Townsend A.J., Goldsmith M.E., Whang-Peng J., Vickers P.J., Poisson R., Legault-Poisson S., Myers C.E., Cowan K.H. (1988). Isolation of the human anionic glutathione s-transferase cdna and the relation of its gene expression to estrogen-receptor content in primary breast cancer. Proc. Natl. Acad. Sci. USA.

[24] Esteller M., Corn P.G., Urena J.M., Gabrielson E., Baylin S.B., Herman J.G. (1998). Inactivation of glutathione s-transferase p1 gene by promoter hypermethylation in human neoplasia. Cancer Res..

[25] Lee W.H., Morton R.A., Epstein J.I., Brooks J.D., Campbell P.A., Bova G.S., Hsieh W.S., Isaacs W.B., Nelson W.G. (1994). Cytidine methylation of regulatory sequences near the pi-class glutathione s-transferase gene accompanies human prostatic carcinogenesis. Proc. Natl. Acad. Sci. USA.

[26] Gomez D.E., Alonso D.F., Yoshiji H., Thorgeirsson U.P. (1997). Tissue inhibitors of metalloproteinases: Structure, regulation and biological functions. Eur. J. Cell Biol..

[27] Gomez D.E., De Lorenzo M.S., Alonso D.F., Andrade Z.A. (1999). Expression of metalloproteinases (mmp-1, mmp-2, and mmp-9) and their inhibitors (timp-1 and timp-2) in schistosomal portal fibrosis. Am. J. Trop. Med. Hyg..

[28] Bachman K.E., Herman J.G., Corn P.G., Merlo A., Costello J.F., Cavenee W.K., Baylin S.B., Graff J.R. (1999). Methylation-associated silencing of the tissue inhibitor of metalloproteinase-3 gene suggest a suppressor role in kidney, brain, and other human cancers. Cancer Res..

[29] Chen K., Sawhney R., Khan M., Benninger M.S., Hou Z., Sethi S., Stephen J.K., Worsham M.J. (2007). Methylation of multiple genes as diagnostic and therapeutic markers in primary head and neck squamous cell carcinoma. Arch. Otolaryngol. Head Neck Surg..

[30] Stearns V., Yamauchi H., Hayes D.F. (1998). Circulating tumor markers in breast cancer: Accepted utilities and novel prospects. Breast Cancer Res. Treat..

[31] Pathak K.A., Khanna R., Khanna H.D., Khanna S., Gupta S., Khanna N.N. (1996). Carcinoembryonic antigen: An invaluable marker for advanced breast cancer. J. Postgrad. Med..

[32] Das P.M., Singal R. (2004). DNA methylation and cancer. J. Clin. Oncol..

[33] Raju U L.M., Sethi S, Qureshi H, Wolman SR, Worsham MJ. (2006). Molecular classification of breast carcinoma in situ. Curr. Genomics.

[34] Schouten J.P., McElgunn C.J., Waaijer R., Zwijnenburg D., Diepvens F., Pals G. (2002). Relative quantification of 40 nucleic acid sequences by multiplex ligation-dependent probe amplification. Nucleic Acids Res..

[35] Kunjoonju J.P., Raitanen M., Grenman S., Tiwari N., Worsham M.J. (2005). Identification of individual genes altered in squamous cell carcinoma of the vulva. Genes Chromosomes Cancer.

[36] Worsham M.J., Pals G., Schouten J.P., Miller F., Tiwari N., van Spaendonk R., Wolman S.R. (2006). High-resolution mapping of molecular events associated with immortalization, transformation, and progression to breast cancer in the mcf10 model. Breast Cancer Res. Treat..

[37] Nygren A.O., Ameziane N., Duarte H.M., Vijzelaar R.N., Waisfisz Q., Hess C.J., Schouten J.P., Errami A. (2005). Methylation-specific mlpa (ms-mlpa): Simultaneous detection of cpg methylation and copy number changes of up to 40 sequences. Nucleic Acids Res..

